# Supplementary material for: Caregivers’ and nurses’ perceptions of the Smart Discharges Program for children with sepsis in Uganda: A qualitative study
Source: PLoS One. 2024 Oct 2;19(10):e0307089. doi: 10.1371/journal.pone.0307089 (PMC11446420; doi:10.1371/journal.pone.0307089)
Supplement: S2 File — (DOCX) [file pone.0307089.s002.docx]

**Supplementary file S2: Focus group discussion guide for the parents/caregivers**

Interview Date: __________________

Smart Discharge Site
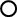
 Mbarara Regional Referral Hospital


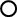
 Holy Innocents Children's Hospital


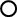
 Jinja Regional Referral Hospital


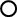
 Masaka Regional Referral Hospital

1. Opening question: What informed the decision to bring your children to the hospital when your children got sick?
2. How did you feel about the discharge process?

- Did you feel prepared for the discharge process?
- How did you feel about the education that you received during discharge?
- Did you learn anything that you felt was helpful?
- Describe the aspects of education that you felt were very important you
- Are there aspects of counselling education that you felt were less important? And why?
- For those whose children were previously admitted: How did it compare to prior discharges?

1. After discharge, some of the children could have recovered, some could have died, and others re-admitted. Describe your thoughts on why the things could have caused such circumstances

- You could ask for scenarios as they describe the causes
- Describe what you think can be the solutions to these causes that you have described?

1. Tell me about the facilities to which you were referred (procedures for referral)

- Was the referral discussed at discharge with the caregiver?
- Were you comfortable with the choice of facility and if not why?
- What is the estimated distance from the facility? Had you ever been to this facility before? How do people travel to this facility?
- If they didn’t go to the referred facility, probe why they didn’t go

1. Please tell me about the follow-up that your child received after you were discharged

- Caregiver’s experience during your referral visits. How did it go?
- How were you treated during the visits by the healthcare provider at the facility? (did they appear to care? Explore care providers’ attitude, communication, etc.
- Education and advice given to the caregiver to improve the care of the child during the post-discharge period.
- How much time spent at facility, and any facility barriers related to the child’s care such as limited time, long queues, stock outs, provider attitude, absence of staff.

1. Some caregivers reported that they had challenges completing the referrals. What is your opinion on this?

- For those who completed the referral, how did you manage to do that? (things that enabled you to complete the referrals (ex. the child was would still show signs, the caregiver fear of the child’s past condition, support from the spouse, the provider emphasized the importance of completion, etc.).
- For those who didn’t complete, please explain what could be the barriers to completing the referral visits as recommended by the healthcare provider? (ex. costs, the child was well, long distance, etc.).
- How do you think these could be addressed?

1. At discharge, you were brought home educational materials. Describe how you used these materials. Did they work for you? Did you like the materials? If yes what aspects?

- How have the educational materials and the helped you to improve care for your child? (if the child is alive). E.g. improved nutrition, best hygiene practices, understanding warning signs, need for health seeking, etc.) (For the children who recovered)
- What concerns do you have about these materials? And how can they be improved
- Probe for the general caregivers’ perception regarding the SD approach (simplicity, convenience, complexity, do the staff care? Are they friendly? Knowledgeable, etc.).

1. Describe how the smart discharge program changed your approach in terms of inclusion of the father/husband in post discharge care and health seeking.

- If father/husband got involved, describe the things that they did to support the mother care for the child (e.g. accompanying the mother to the facility, giving advice) if they never used to do so.
- How did the husband/father involvement contribute to the child’s improved health? E.g. health seeking, better understanding of recovery, better meals for the child etc.
- Describe the challenges you encountered while caring for your child during recovery after discharge.
- In cases where the child became sick again or died, describe what prevented you from giving adequate care for your child AFTER discharge.
- Describe what your thoughts about the causes of these barriers within the Smart Discharges program (environmental, financial, social, etc.)
- Explain your thoughts on what Smart Discharges Program could have done for/with the family to address these barriers (especially if the child died, re-admitted).
- Explain what more you think the project should do to address these challenges mentioned above.

End
