# Supplementary material for: Caregivers’ and nurses’ perceptions of the Smart Discharges Program for children with sepsis in Uganda: A qualitative study
Source: PLoS One. 2024 Oct 2;19(10):e0307089. doi: 10.1371/journal.pone.0307089 (PMC11446420; doi:10.1371/journal.pone.0307089)
Supplement: S3 File — (DOCX) [file pone.0307089.s003.docx]

**Supplementary file S3: Interview guides for nurses**

Facility………………………………………Date………………………………………………..

Sex of participant…………………………....Age …………………………………………….

1. Tell me how you are involved in providing care and support to caregivers of children suffering from severe infections in this facility? (describe what your role involves in the Smart Discharges Program (SDP))

- Probe: how do children with severe infections come in the facility, what happens during risk assessment, caregiver counselling and education during admission, counselling at discharge, issuing of the post-discharge referral.

1. As a nurse, tell me about your experience in providing assessment, counselling and caregiver education to promote compliance to the Smart Discharges Program at your facility
   - Probe: Tell me more about the risk assessment, how easy or difficult is it, how long does it take, etc.
   - Probe: Tell me about counselling/educating caregivers when the child is in hospital and at discharge (do they find it easy to communicate the info to caregivers) what challenges do they face in understanding, what challenges do you face in communicating the concepts, etc. How do the parents react to these materials (bag, notebook, instructions)? How do you feel they can help in caring for the child?
   - Probe: are there instances when you aren’t able to ably perform the discharge process as instructed? What are the barriers? What might improve the process
2. In your experience, which components of the Smart Discharge Program package (risk assessment, counselling, educational materials, post-discharge referral for follow-up) are working well.

- Probe: how do you define working well --- why?
- Probe: Be more open-minded and give them the opportunity to speak more broadly about the other aspects of their engagement in the Smart Discharges Program, including communication among themselves.
- Are there components of this program that you think may not be working well to improve discharge of children admitted for sepsis? How do you define not working well and why?

1. In your opinion, how would you describe the support that you are currently receiving in terms of implementing the Smart Discharges Program for children who have suffered from severe infections?

- Probe if they are supported well enough to provide this service in terms of training, equipment, supervision etc.
- Probe: How would you define well supported/not well supported? (depending on the previous response)

1. As a health professional working with caregivers of children who have suffered from severe infections, can you describe for me the potential benefits of this program to the families of children with severe infections
2. Based on your experience working with caregivers of children admitted and discharged for severe infections, what are some of the challenges that you face in terms of caregivers adhering to counselling and education that you provide?
3. In your opinion what would be some of the potential barriers preventing caregivers from accessing care even when they are enrolled in the SDP?
4. In your opinion, describe the aspects of the SDP that you feel need to be improved

Probe: for why and how the components of SDP can be improved

End
