# Supplementary material for: Caregivers’ and nurses’ perceptions of the Smart Discharges Program for children with sepsis in Uganda: A qualitative study
Source: PLoS One. 2024 Oct 2;19(10):e0307089. doi: 10.1371/journal.pone.0307089 (PMC11446420; doi:10.1371/journal.pone.0307089)
Supplement: Attachment — Submitted filename: Response to Reviewers from PGPH-D-23-01267.docx [file pone.0307089.s004.docx]

December 15, 2023

Dear Editors and Reviewers,

Thank you for reviewing our recently submitted manuscript (PGPH-D-23-01267; PONE-D-23-37812), “Caregivers’ and nurses’ perceptions of the Smart Discharges Program for children with sepsis in Uganda: A qualitative study”. Below is our point-by-point response to the original reviewers’ comments from our submission to PLOS Global Public Health. We know that this process takes time, and we appreciate the thorough feedback from reviewers that have strengthened our manuscript.

Sincerely,

**Dr. Matthew O. Wiens**, PharmD, PhD

Assistant Professor

Department of Anesthesiology, Pharmacology & Therapeutics | Institute for Global Health

The University of British Columbia | BC Children's Hospital and BC Women’s Hospital + Health Centre

938 W 28th Ave | Vancouver BC | V5Z 4H4 Canada

Phone 604 997 7753 | Email [matthew.wiens@bcchr.ca](mailto:matthew.wiens@bcchr.ca)

**COMMENTS:** PGPH-D-23-01267

**TITLE: Caregivers’ and nurses’ perceptions of the Smart Discharges Program for children with sepsis in Uganda: A descriptive qualitative study**

**Response to Reviewer 1**

**Reviewer Comment 1:** Lines 56–88: The introduction provides a clear overview of the global burden of sepsis among children, especially in LMICs. Consider adding a brief overview of the burden and management of sepsis in Uganda. This will enhance the context of the study.

**Response 1:** Thank you for the suggestion to incorporate additional detail regarding sepsis management into the introduction. While we agree that such details represent important content, after much consideration we feel that such an addition would detract from the main scope of this paper, which reflect management during/after discharge. However, we have added a sentence into the introduction regarding the fact that sepsis guidelines currently do not incorporate guidance about discharge/post-discharge care. This is a major limitation of current guidelines, and one which needs to be rectified in future iterations of sepsis guidelines for LMIC settings.

We have added the following sentence:

*Current sepsis guidelines have not sufficiently incorporated post-discharge care, leaving health workers with limited guidance.*

**Reviewer Comment 2:** Write the complete sentence when "Smart" is used for the first time (Signs, Medications, Appointments, Results, and Talk with Me), and then you can use the abbreviation in the second use and so on.

**Response 2:** In the context of “Smart Discharges” the word Smart is not an acronym and does not stand for Signs, Medications, Appointments, Results, and Talk with me.

**Comment 3:** The authors have identified the lack of exploration of caregivers' and nurses' perspectives as a research gap that this study aimed to address. Line 84: There is a need to expand on why understanding caregivers' (service consumers) and nurses' (service providers) experiences and perceptions is crucial and how their insights could contribute to the improvement of the Smart Discharges Programme’s effectiveness. This will highlight the significance of this qualitative study. Also provide a reference for the Smart discharges protocol.

**Response 3:** Thank you for this comment, the following has been added to address this (lines 86-90):

*At this time, the perceptions and experiences of the parents/caregivers and nurses participating in this program have yet to be explored. The caregivers are the key users of the program and the nurses are responsible for the delivery of the program. As such, their experiences are crucial to understanding opportunities for program improvement and future scaling.*

The following reference has been added to the introduction, which outlines the Smart Discharges protocol:

Wiens MO, Kumbakumba E, Larson CP, Moschovis PP, Barigye C, Kabakyenga J, et al. Scheduled Follow-Up Referrals and Simple Prevention Kits Including Counseling to Improve Post-Discharge Outcomes Among Children in Uganda: A Proof-of-Concept Study. Global Health: Science and Practice. 2016;4(3):422-34.

**Reviewer Comment 4:** Generally, the methodology section is well described. However, some parts need to be clarified:

Study setting: The authors should provide a rationale for selecting participants from the four referral hospitals. Are the four hospitals representative of health facilities offering the Smart Discharge Program (SDP) or the broader healthcare system in Uganda? Were there implications for transferability? If so, that should be acknowledged in the limitations section.

**Response 4:** The following rationale was added as to why participants were selected from the four referral hospitals in this study (lines 104-107):

*The study was carried out at four hospitals in Uganda, including three public regional referral hospitals (RRH) and one private-not for profit hospital (Table 1). These were the first four hospitals to implement the Smart Discharges Program in Uganda, which has expanded to further hospitals since the time of data collection.*

The limitations around the transferability of study findings have been added to the limitations section (lines 482-484).

**Reviewer Comment 5:** Inclusion criteria: Lines 111–114: Please justify the 2-month inclusion criteria for nurses and caregivers – is this the timeframe within which nurses and caregivers are likely to have had significant exposure to the SDP? Also describe how the participants were identified by the Social Scientist (OK). From medical records maintained at the hospitals or SDP records?

**Response 5:** The rationale for the 2-month timeframe for caregivers to be included in the study and how participants were identified by OK has been added to the methods section (lines 114-118):

*Caregivers were identified using Smart Discharges study records. They were eligible if their child had been enrolled into the Smart Discharges program and had been discharged at least two months prior to recruitment. Recruitment of caregivers was conducted after two months to allow them to share their experiences with follow up appointments and applying discharge education at home.*

**Reviewer Comment 6:** How were the FGD and the semi-structured interview guides developed? Were they based on expert opinions, previous research, existing literature, or standardised tools?

**Response 6:** More detail on how the FGD and the semi-structured interview guides were developed has been added to our Methodology under the Data Collection section (lines 132-134):

*Topics in the interview and FGD guides were developed by senior investigators (MOW, NKM, STJ) who have expertise in the field of pediatric sepsis.*

We also improved the limitations section, to highlight the limitations within the development of the data collection tools (478-480):

*Secondly, piloting of data collection tools and use of a conceptual framework were not components of the design of the interview guides, which may have decreased the validity of the instruments used to collect data*.

**Reviewer Comment 7:** Line 174: Include a rationale for conducting separate FGDs for fathers and mothers/other female caregivers. Did the perspectives and experiences differ between the two groups? Why was capturing their unique viewpoints important for the study's objectives? Did this improve the quality of the data collected?

**Response 7:** In Uganda, fathers and mothers typically play very different roles in the care of children. Thus, it was deemed important to separate father and mothers into different focus groups. Furthermore, to make the discussions more comfortable for participant, especially the women, we decided that separate focus group discussions would be preferred.

**Reviewer Comment 8:** Lines 131-133 provide an explicit description of how the researchers tracked, monitored, and determined saturation/richness of data across FGDs and interviews. Currently, the description is not adequate. This will help provide justification for the relatively small sample size of 4 FGDs and 8 interviews conducted and underscore reliability of the qualitative findings.

As an illustration: To track and monitor saturation, the researchers combined sampling, data collection, data analysis and assessment rather than treating them as separate stages in a linear process (Moule, Aveyard & Goodman). The researchers went through multiple sequential analysis rounds until no new information or themes were generated. Saturation was subjectively determined by the researchers to have occurred after conducting four FGDs and eight interviews.

Moule Pam, Aveyard Helen and Goodman Margaret. Nursing Research: An Introduction. 3rd Edition. 2017. Sage Publications. London/Losangeles/New Delhi/Singapore/Washington DC.

**Response 8:** We agree that our previous description was lacking important details, and have improved our analysis section to justify our sample size. See our Data Analysis Section (lines 143-160).

**Reviewer Comment 9:** Line 137: The FGD and interview guides were not pre-tested prior to use, and no repeat interviews were done. Provide valid reasons why (QOREC item 17). Was this because of limited participant availability, time/resource allocation, high experience levels of the researchers who designed the guides, etc.?

**Response 9:** The FGD and interview guides were not pre-tested prior to use and no repeat interviews were done due to budget and logistical constraints at the time of data collection. This was added to the methods section (lines139-141) and the limits of this were added to the limitations section.

**Reviewer Comment 10:** Also participant checking was not done. Please provide a valid reason why (QOREC item 28). Participant checking evaluates the credibility of the results by testing the data, analytic categories, interpretations, and conclusions with representatives of the group from whom the data were originally obtained. How was this gap in credibility-checking addressed?

**Response 10:** Member checking was not done due to the same budgetary and logistical constraints that limited pre-testing of the FGD and interview guides. For example, we could not reliably follow up with participating caregivers because they reside in distant and rural locations. Validity of study results was maximized through our data analysis process, which involved open coding by two independent coders, followed by inductive and deductive identification of themes and subthemes, and discussion across the study team. These steps are described in detail in lines 143-160.

**Reviewer Comment 11:** Regarding triangulation of data sources – how was the data from FGDs and interviews used to form the themes? Did the researchers compare, contrast and integrate themes from FGDs and interviews? If so, how were the themes from FGDs and interviews integrated to identify areas of convergence and divergence? This will help the reader to understand the themes and structuring of the study results.

**Response 11:** The data analysis section has been updated to answer the above questions (below excerpt from lines 144-160):

*Using a thematic analysis approach, initial open coding of transcripts was done by two investigators (JB and OK)… Initial codes were categorized both within and between sources. From this, the two investigators (JB and OK) independently created the initial coding framework, deductively using the FGDs/interview guide topics and inductively to identify emergent themes and subthemes. The coding framework was revised and refined through discussion between JB, OK, and MOW. After development of the coding framework and initial coding, themes were proposed, and discussed between JB, OK, and MOW who jointly agreed on the study themes.*

**Reviewer Comment 12:** The Results section is well organised, featuring clear headings and subheadings. It effectively presents the combined perspectives of SDP service providers and service consumers. However, the authors should pay attention to the following areas:

Lines 178–180: If available, the authors should add more demographic information to Table 2 (e.g., level of education and employment status) to enhance the description of the studied population.

**Response 12:** Though we do have some of these data for mothers, we do not have these data for nurses and for fathers/male caregivers. While we agree that such data may be useful, we unfortunately cannot report these. Regardless, we do believe that the responses speak for themselves and though we perhaps cannot fully contextualize them form a social and economic perspective, we feel these results remain valuable.

**Reviewer Comment 13:** In alignment with the descriptive phenomenological design and objectives of the study, it is essential for the authors to ensure a balanced representation of viewpoints and quotes from both caregivers and nurses across all themes and subthemes. E.g.

- Facilitators to follow-up care after discharge: Line 220-221 and 222-226 Consider including quotes from caregivers.
- Barriers to follow-up care post-discharge: 267-269 Consider providing quotes from caregivers.

**Response 13:** Within both the facilitators and barriers to follow up care after discharge sections, quotes from caregivers have been included to ensure that there is balanced representation of viewpoints.

**Reviewer Comment 14:** Consider including information on caregivers’ and nurses’ recommendations for improvement of SDP in the results section. Files S2 and S3 included questions to elicit participants' perspectives on how to address challenges or barriers and/or improve the SDP. Ref File S2 &S3.

**Response 14:** We appreciate the reviewers careful reading of the supplementary files. Though we did capture some information on how to further improve the SDP, we did not feel we had sufficient space in this present manuscript to include it, given its current length. We plan to utilize these data for a separate paper in the future, once we have had a chance to conduct further qualitative work in this area.

**Reviewer Comment 15:** The discussion effectively summarises the key findings of the study, highlighting the positive impact of the SDP. I strongly recommend that the authors enrich the discussion by providing or referencing evidence-based practices in the approaches they propose to strengthen post-discharge care. Also nurses’ and caregivers’ recommendations for improvement should be considered. E.g. Lines 418–420: community engagement – the authors propose strengthening educational engagement at the community level, such as by collaborating with schools, religious institutions, and community groups. How could these partnerships be formed and sustained? Reference the available practice/literature.

**Response 15:** Thank you for this important comment. We agree that such partnerships may be critical in the future success of programs such as ours. However, we have not yet delved into the best strategy to scale building such partnerships. The focus of this paper was not to develop these strategies but rather guide future efforts to strengthen our programs. We are beginning to find ways to do this, but do not wish to add further length to an already lengthy paper. Our intention is to conduct a systematic literature review in addition to consulting experts in Uganda, to determine the best course of action for partnership engagement.

In order to address the example of community engagement, we referenced the below paper in the discussion. This article shows that community level interventions can be effective; however, it is not yet evaluated in the context of pediatric post-discharge care.

Reference: 28. Black RE, Taylor CE, Arole S, Bang A, Bhutta ZA, Chowdhury AMR, et al. Comprehensive review of the evidence regarding the effectiveness of community-based primary health care in improving maternal, neonatal and child health: 8. summary and recommendations of the Expert Panel. Journal of global health. 2017;7(1):010908.

**Reviewer Comment 16:** 423–425: HCW education and paradigm shift - elaborate on how this education and paradigm shift could be effectively integrated into the existing healthcare system. Are there specific training programmes, guidelines, or policies that could be used or developed?

**Response 16:** Thank you for highlighting that we should consider how this education and paradigm shift could be effectively integrated into the existing healthcare system. In response to the comment, we have added the suggestion that policy/guidance such as the Integrated Community Case Management (iCCM) needs to be improved to include children who were recently discharged from hospital (lines 439-442). The Smart Discharges research team is presently working with the Ministry of Health in Uganda to update iCCM to include “care of the discharged child”.

**Reviewer Comment 17:** 431-440: Male community member engagement **-** how can this engagement be developed, maintained and expanded in diverse cultural contexts?

**Response 17:** While we agree that male community member engagement is important and is indeed a key finding, expanding on how this can be achieved is beyond the scope of this paper. This topic is clearly very broad and we, unfortunately, do not have wish to detract from the main findings, which could result in a loss of readership. Future papers will focus on aspects related to strategy, which includes other aspects as well, such as building partnerships with community groups.

**Reviewer Comment 18:** 441-457: Financial constraints - in addition to telemedicine and the involvement of CHWs, you could also identify and reference other practical solutions, such as subsidised transportation for those living in areas with long distances to a health facility or innovative funding mechanisms for post-discharge care.

**Response 18:** We do also agree that other practical solutions, such as subsidised transportation and innovate funding mechanisms are excellent suggested solutions. We feel that expanding further on this goes beyond the scope of the paper. We have however, added a sentence suggesting that telemedicine or CHW follow-up could be practical solutions in these contexts (lines 461-463):

*For caregivers with constrained resources, a more cost-effective alternative to improving post-discharge care, such as the use of telemedicine or community health workers for post-discharge follow-up, could have a substantial impact.*

**Reviewer Comment 19:** Lines 459-463: Consider leaving out this limitation. Clearly, eliciting the experiences of older children was not within the study’s scope and objectives.

The authors should consider including the limitations inherent in qualitative research and how they were managed and mitigated.

**Response 19:** The limitation on eliciting the experiences of older children has been removed, and the limitations section has been improved to include limitations that are inherent in qualitative research as well as some of the methodological limitations of this study (i.e. interview guides were not pre-tested). See lines 474-484 of the manuscript.

**Responses to Reviewer #2:**

**Reviewer 2 Comments 1:** This qualitative study evaluates caregiver and nurse perceptions of the Smart Discharges Program, which identifies children at high risk of clinical decompensation post-discharge and aims to provide enhanced post-discharge care. The study is well designed and executed, and given the article discusses global health delivery and touches on the social and behavioral aspects of healthcare, it is relevant for the readership of this journal. Comparable programs could be implemented in similarly-resourced settings. To further strengthen the quality of the paper, the authors could provide more clarifications and details around their methodology. Please see my comments below.

- The definition of sepsis does not entail organ dysfunction- this is the definition of severe sepsis. Consider editing or removing the first line of the abstract.
- I would suggest modifying the organization of the results in the abstract. For example, the authors nicely summarize their results using themes and subthemes in the manuscript body. On first read, it was difficult to tell if they were defining the key themes or listing sub-themes, and the reader has to connect each elaborative sentence to the stated theme. The authors could be explicit that they are listing key subthemes and list them under each numbered key theme.

**Response 1:** Thank you for suggesting re-organizing the abstract. The structure of the abstract has been improved to provide clarity. With regards to the definition of sepsis, our intent was to define sepsis as a concept, but not provide the criteria for sepsis. However, we have slightly modified our conceptual definition of sepsis to: “Sepsis arises when the body’s response to an infection injures its own tissues and organs” based on the Global Sepsis Alliance definition.

**Reviewer 2 Comments 2**:
- The introduction is well written with appropriate flow and scope.
- I appreciated the referenced material. It might be helpful to include more details about the Smart Discharges Program within the intro text to give readers more context. For example, the authors might briefly summarize the predictive factors for post-discharge mortality used to identify patients enrolled in this program (e.g., MUAC, SpO2, BCS, serostatus, etc.) and how front-line health workers “streamline a path for follow-up care” (e.g., referrals, follow-up texts, etc.).

**Response 2:** Further details about the Smart Discharges Program have been added to the introduction as shown below. In an effort to keep the introduction fulsome, yet concise, we have also directed readers to a previous publication which provides a higher level of detail and included a citation to a dataset that includes the Smart Discharges Post-Discharge Care Counselling Guides. An excerpt of these changes is below (lines 73-83):

*The Smart Discharges Program uses a mobile health (mHealth) platform to identify children at high risk of post-discharge mortality for enhanced post-discharge care. The model used in Smart Discharges predicts risk of future mortality using patient demographics, anthropometric measurements, and clinical indicators collected on admission (14-17). This innovative program provides an opportunity for front-line health workers in resource-poor environments to identify vulnerable children and to streamline a path for follow-up care during the post-discharge period (17). Counselling and discharge planning target factors that are known or assumed to contribute to post-discharge mortality, including clinical, social, and system considerations (18). While all participants receive enhanced discharge counselling, high-risk children receive down-referrals to community health facilities for follow-up, which is detailed in a previous publication (12).*

**Reviewer 2 Comments 3**:
- Some details could be better organized within the subsections of the Methodology section to reduce redundancy. Exs: “Data were collected through in-person focus group discussions (FGDs) with caregivers of pediatric patients enrolled in the Smart Discharges Program and individual, semi-structured interviews with Smart Discharges Program nurses…” could be outlined in the recruitment/data collection sections. “Interviews were conducted in person in private rooms within the pediatric wards at the study sites” could be mentioned in the Data Collection section. Etc.

**Response 3:** Thank you for these recommendations to reduce redundancy and improve the flow of the methodology section. We have incorporated your feedback, among other improvements to the methodology section which are detailed throughout this response to reviewers.

**Reviewer 2 Comments 4**:

- My impression on seeing the results is that this is not a phenomenological study as much as it is a general interpretivist, or basic qualitative, study. Basic qualitative studies describe the lived experience [e.g., as a caregiver, I bathe my mother, I feed her, etc.] and phenomenology describes the essence of the experience [e.g., as a caregiver, the experience is heart wrenching and isolating, etc.]. Phenomenology gets at emotions/feelings/interfaces and tends to be deeper with regards to expressing intense human experiences.

**Response 4:** We agree and have removed reference to this being a phenomenological study.

**Reviewer 2 Comments 5**:

- I would be interested in more information on the study setting to know if the Smart Discharges Program would be applicable to settings in which I’ve worked. Who are the front-line providers discharging patients and is there pediatrician oversight? If available, what is the prevalence of co-morbid disease among the patient populations which may predispose patients to sepsis-related mortality? Are the referral hospitals or the district hospitals which feed into the referral hospitals associated with satellite clinics? What resources are available to patients prior to discharge (in-hospital pharmacy, routine social work consultation, translators for patient education as needed, formalized referral processes, etc.)?

**Response 5:** We agree that it is essential to have information on the Smart Discharges Program that allows readers to know if the Program would be applicable to settings the readers are working in, and which includes detailed answers to the questions above. However, addressing these inquiries is beyond the scope of this paper. To provide more details we have added a previous publication within the introduction, which shows how we did the intervention (lines 69-70):

*Such interventions may form critical components necessary to improve the long-term survival of children with sepsis (12)*

Additionally, a separate study is underway to explore the effectiveness of the Smart Discharges Program, and will hopefully address some of these above questions once published.

**Reviewer 2Comments 6**: - Did access to a phone influence enrollment in the program and/or recruitment?
- What did you do when potential recruits refused participation or were not reachable by phone?

**Response 6:** Approximately, only 1% of participants did not have access to a phone, so it did not have a substantial impact on enrollment in the program and recruitment. If participants did refuse participation, then they were not enrolled into the program, which did occur occasionally.

**Reviewer 2 Comments 7:** - It would be helpful if the authors stated the conceptual framework used to design the interview guides and interpret the data. The authors state that the guides were not “pre-tested,” which I assume means the guides were not piloted or tested with cognitive interviews. If piloting/use of a conceptual framework were not components of the design of the interview tools, that should be mentioned in the limitations section as a potential factor that decreases the validity of the instruments used to collect data.
- Did the authors analyze data and conduct interviews concurrently to ensure thematic sufficiency?
- Many of the prompts asked in the interview guides are not open-ended questions which could potentially limit the interviewer’s ability to understand the construct being studied and interfere with reaching thematic sufficiency
- Was there a single round of coding or were multiple rounds conducted (if so, how many rounds)? How did the investigators who independently created the initial coding framework handle discrepancies?

**Response 7:** Thank you for these comments/questions, which will help strengthen our methodology section.

Piloting or pre-testing were not components of the design of the interview tools, and this limitation has been added to the limitation section of the manuscript. The interview guides were developed by the senior investigators of this study, who have expertise in pediatric sepsis, the smart discharges program, and the study context.

Using a thematic analysis approach, initial open coding of transcripts was done by two investigators. Initial codes were categorized both within and between sources. From this, the two investigators (JB and OK) independently created the initial coding framework, deductively using the FGDs/interview guide topics and inductively to identify emergent themes and subthemes. The investigators handled discrepancies, and revised and refined the coding framework through discussion amongst themselves and with the Principal Investigator. When necessary, the investigators would go back to the data to enrich the discussion. The above details have been added to the methodology section (lines 144-160).

With regards to the prompts, we agree that some were not open ended, which may have limited the interviewer’s ability to understand the construct.

**Reviewer 2 Comments 8**: - The statement, “two researchers (JB and OK) analyzed the data and discussed their findings to produce the coding framework,” does not address reflexivity. Did the researchers formally examine their own feelings, reactions, and motives participating in the research?
- As stated above, I would consider providing additional information about the study setting to improve transferability.

**Response 8:** Within the Trustworthiness section the sentence following the one quoted above, “They discussed potential biases, questions, and concerns, allowing for a sharing of perspectives (24)” addresses reflexivity. The limits of the transferability of this study have been added to the limitations section.

**Reviewer 2 Comments 9**:
- Given that the authors purposively sampled for certain characteristics (readmission, post discharge mortality, adherence and non-adherence to prescribed post-discharge follow up), I was surprised that only 1 of 30 caregivers experienced a child with post-discharge mortality. This is a potential limitation of the study. The authors should list the number of caregivers completing follow-ups in the following line (line 184): “Out of the 30 caregivers, reported completing all three follow up appointments…”

**Response 9:** This comment brings up an excellent point. The following was added to our limitation section to address this: “Within the study’s participants, one caregiver had experienced the post-discharge death of their child. Therefore, the experiences with the most adverse and serious outcome was not well captured by the sample” (lines 480-482).

**Reviewer 2 Comment 10:** - Interesting data and very helpful. My perception is that the quotes could be shortened and reduced in number without losing information.

**Response 10:** Thank you for this suggestion, some of the quotes were shortened when it was possible to do so without losing meaning.

**Reviewer 2 Comments 11**:
- The authors did a nice job of contextualizing their findings in the existing literature, providing perspective on how their study findings add to the literature, and offering recommendations for similar programs.

- Of 32 references, 9 (28%) are greater than 5 years old, which is reasonable

**Response 11:** Thank you, we appreciate this positive feedback.

**Response to Reviewer #3:**

**Reviewer 3 Comments 1:** This is a qualitative research study examining perceptions about the Smart Discharges program. The study included focus groups with parents/guardians of patients enrolled in the study and individual interviews with nurses. The study took place in 2019 and my understanding is that this qualitative component occurred while Smart Discharge was being studied as well.

I would have liked more information on Smart Discharge. It was unclear to me what program these patients/families were enrolled in or what training the nurses involved received prior to/during the program - making it challenging as the reader to understand how it might impact them. At one point they mention SMS messages - but do not say if these were automatic or by a nurse, phone calls - but it doesn't state who calls the families, and they mentioned "complementary aspects" (line 200, line 223) of the study but do not describe them well. The discharge instructions are highlighted throughout this manuscript but it is unclear in this paper what the education around discharge instructions might be in the Smart Discharge program. The authors have a sentence addressing the number of follow-up appointments attended by families (line 184) but it is missing the number ("Out of the 30 caregivers, reported completed..."). The authors also fail to mention when these focus groups occurred in relationship to their enrollment in program/discharge from the hospital which may affect their recall bias. Furthermore, I imagine that the data regarding the Smart Discharge program - including in-hospital and post-discharge mortality numbers - and its impact on post-sepsis care may be available but it is not mentioned here. Whether or not the level of participation these focus group participants had in this program was typical of the Smart Discharge program is not mentioned.

**Response 1:** More details and previous publications on the Smart Discharges Program have been added to the introduction to provide more information for readers. This includes the following manuscript that describes the intervention:

Wiens MO, Kumbakumba E, Larson CP, Moschovis PP, Barigye C, Kabakyenga J, et al. Scheduled Follow-Up Referrals and Simple Prevention Kits Including Counseling to Improve Post-Discharge Outcomes Among Children in Uganda: A Proof-of-Concept Study. Global Health: Science and Practice. 2016;4(3):422-34.

We have also included a citation to a dataset that includes the Smart Discharges Post-Discharge Care Counselling Guides:

Uganda Ministry of H, Komugisha C, Kenya-Mugisha N, Wiens M. Counselling Guides for Post-Discharge Care ~ Smart Discharges. V1 ed: Borealis; 2022.

This paper is part of a much larger program of study, which does include data regarding in-hospital and post-discharge mortality. Given the scope of the paper and previous publications, these are not detailed in this manuscript. The following publication has been cited, which provides the most comprehensive overview of this data to date:

Wiens MO, Bone JN, Kumbakumba E, Businge S, Tagoola A, Sherine SO, et al. Mortality after hospital discharge among children younger than 5 years admitted with suspected sepsis in Uganda: a prospective, multisite, observational cohort study. The Lancet Child & Adolescent Health. 2023;7(8):555-66.

**Reviewer 3 Comments 2:**
The study was carried out at four hospitals in Uganda. Three of these hospitals appear to be quite far apart - the fourth (Holy Innocents Children's Hospital) is close to Mbarara RRH. The authors could consider describing - perhaps in the discussion - whether or not the results of this study represent a broad swathe of Uganda based on the hospitals included. Note, I believe there may be a typo in the estimated total annual pediatric admissions at Holy Innocents Children's Hospital - it is orders of magnitude smaller than the other hospitals despite having a similar number of pediatric bed. There is also a typo (I believe) in line 466 in the conclusions mentioning that all of these hospitals are in the same county (rather than country).

**Response 2:** Thank you for identifying the error in estimated pediatric admissions, we have updated Table 1 to reflect accurate estimates of hospital pediatric admission capacity during the time of data collection.

The typo in line 466 has been corrected.

**Reviewer 3 Comments 3:**
It is unclear what level of adherence (or really what adherence to the Smart Discharge program might mean) the patients/families in this program had to the Smart Discharge. If this is available, the authors should consider including it as it would certainly affect their perceptions of the program.

**Response 3:** We have softened the language from adherence to simply applying the discharge care instructions, which are outlined in references 12 and 18, and attendance at post-discharge follow up visits (lines 204-206):

*Follow up care after discharge was defined as applying the discharge care instructions as applicable to their child and attendance at post-discharge follow up visits*.

**Reviewer 3 Comments 4:**
This is a qualitative research study. It is not clear to me how many of the investigators coded each interview, whether some interviews were coded by more than one author, no kappa is reported. The paragraph on 'trustworthiness' in the Methods section is particularly confusing to me. Rather than describing their own methods, they appear to be citing other articles on how a qualitative study should be conducted. I do not think I could repeat their study based on the explanation of their methods provided. I defer to those reviewers with more experience in qualitative research to evaluate these methods. The authors do not mention whether focus groups included people from various sites enrolled or only a single site - i.e. it is unclear if all of the members of the male focus group came from a single site or not.

**Response 4:** Throughout the methodology, author initials have been added to indicate which authors and how many were involved in each process. We did not report kappa as its often not done in qualitative studies of this type.

The methods section has been revised and improved to provide clarity on how the study was conducted. The articles that are cited that describe how a qualitative study ought to be done were used to guide the methodological rigor of this study.

**Reviewer 3 Comments 5:**

I found the themes they uncovered interesting. In particular, I had to wonder if the study engaged the nurses in such a way that they might have found more time to speak to the family which in turn may have led to both more empathy for the patients/families and perhaps a sense of empowerment that their discharge instructions could indeed be an agent of change (quotes on line 212, 243). On line 272, the authors state, "Participants noted that appreciating the relevancy and the timing of counselling [sic] was important." It seems that participants that the relevancy and timing of counseling was important - not appreciating it. Furthermore, counseling is spelled incorrectly.

**Response 5:** Thank you for these insightful comments. To improve clarity, we have adjusted the wording as shown below (lines 275-279):

*Caregivers and nurses reported that the discharge education provided often resulted in changed caregiver behavior, noting that the relevancy and the timing of the counselling was important. When information was relevant and well timed, caregivers’ knowledge about post-discharge care of their children increased, which facilitated improved care of their recovering child once they left the hospital.*

**Reviewer 3 Comments Response 6:**

I was surprised to read in the conclusions that the Smart Discharges program targets the involvement of male caregivers (line 469-470). It seemed previously in the paper that this was a lucky side effect -- and I wonder if this first sentence in the conclusion is just written in such a way that it makes that confusing. The remainder of the conclusions made by this study are very broad. "Smart Discharges approach is an impactful strategy to improve pediatric post-discharge care..." is a comment that might stem from the primary research study evaluating deaths/repeat hospitalizations in these children. Rather, my takeaway of this study is that it demonstrated that the Smart Discharge program helped engage caregivers, including fathers, and facilitated conversations between nurses and families.

**Response 6:** Thank you for this comment about the conclusion. We have changed the wording in the first sentence to provide clarity, and have incorporated the key takeaways from the study (lines 486-493).
